# Low dose of Bisphenol A enhance the susceptibility of thyroid carcinoma stimulated by DHPN and iodine excess in F344 rats

**DOI:** 10.18632/oncotarget.19434

**Published:** 2017-07-22

**Authors:** Jing Zhang, Xiaochen Zhang, Yanan Li, Zhenzhen Zhou, Chuanlong Wu, Zhiyan Liu, Lanxiang Hao, Shanshan Fan, Fang Jiang, Yan Xie, Ling Jiang

**Affiliations:** ^1^ Department of Endocrinology, Qilu Hospital of Shandong University, Jinan 250012, China; ^2^ Department of Hemodialysis, Heze Municipical Hospital, Heze 274000, China; ^3^ Department of Nursing, Heze Medical College, Heze 274000, China; ^4^ Department of Endocrinology, Laiwu City People’s Hospital, Laiwu 271100, China; ^5^ Department of Radiotherapy, Jinhua Municipal Central Hospital, Jinhua 321000, China0; ^6^ Department of Pathology, Qilu Hospital of Shandong University, Jinan 250012, China; ^7^ Department of Endocrinology, Yancheng First People’s Hospital, Yancheng 224001, China

**Keywords:** Bisphenol A, iodine excess, thyroid carcinoma, estrogen receptor α, PCNA

## Abstract

Thyroid carcinoma (TC) is the most common endocrine neoplasm. The risk of TC as a second primary malignancy (SPM) of breast cancer is significantly increased. Bisphenol A (BPA) is a widely contacted xenoestrogen and increases susceptibility to breast cancer through binding to estrogen receptor alpha (ERα). However, the effect of BPA on thyroid carcinogenesis has not been fully demonstrated. This present study aimed to characterize the effects of BPA on the development of TC using a Fischer 344 (F344) rat model. In this study, we established a TC model using female F344 rats pretreated with N-Bis (2-hydroxypropyl) nitrosamine (DHPN) at a single dose of 2800 mg/kg (the DA group) or without DHPN (the DN group), followed by stimulation with BPA at the level of 250 μg/kg (BPA250) or 1000 μg/kg (BPA1000) and a basic diet containing potassium iodine (KI, 1000 μg/L) for 64 weeks. We demonstrated that the incidence of TC in the BPA250 + KI of DA groups reached the highest at 50%, the incidence of thyroid hyperplasia lesions (including both tumors and focal hyperplasia lesions) in the BPA1000 + KI of DA groups reached 100% (*P* < 0.05). ERα protein and immunochemistry expression was upregulated in the BPA-exposed groups and the immunochemistry scores were positively correlated with PCNA. Thus, the present results indicate that BPA could enhance the susceptibility to TC stimulated by DHPN and iodine excess. ERα is probably involved in the proliferation effect of BPA. BPA or KI alone could not increase TC incidence.

## INTRODUCTION

TC is the most common malignant tumor in the endocrine system and is mainly categorized into papillary, follicular, medullary, and anaplastic TC. Papillary thyroid carcinoma (PTC) accounts for more than 80% of all the pathological types [1]. The risk factors for the morbidity of TC include an excess and/or deficiency of iodine intake [2], radiation exposure [3], sex hormone, and environmental pollutants [4], among others. Tumorigenesis is due to the abnormality of gene alterations and regulations, which leads to unlimited cell proliferation [5]. For the thyroid, focal papillary hyperplasia and C-cell proliferation are common types of thyroid proliferative lesions [6, 7].

Iodine excess usually occurs when the median urinary iodine (MUI) is more than 300 μg/L [8]. A clinical study showed that an excessive iodine state was seen in 66.99% of PTC patients, significantly higher than control group with a ratio of 19.93%, which demonstrated an association between high MUI and thyroid malignancy [9]. A 5-year prospective epidemiological study showed 10 patients were identified and 13 new cases were diagnosed as TC in an excessive iodine intake area, while none was found in both deficient and normal iodine intake areas [6]. An increased incidence of PTC was also found in Hawaii and Iceland, which are both high iodine intake areas [10, 11]. DHPN is a drug with the ability to stimulate thyroid proliferation and convert into adenoma or carcinoma [12]. In animal researches, iodine excess has been reported to promote the proliferation of thyroid follicular cells and eventually result in TC if animals are pretreated with DHPN [2, 13]. Nevertheless, the definite carcinogenesis effect of excess iodine is still controversial; more evidence is needed *in vivo* and *in vitro*.

TC is a hormone-dependent carcinoma [14, 15]. The ratio of females to males is about 3∼5:1. The risk of TC as an SPM of breast cancer is significantly increased and vice versa, suggesting the effect of estrogen could not be neglected [16]. Estrogen could stimulate cell proliferation via ERα [17, 18]. In human TC tissue, elevated expression of ERα has also been reported, especially when coexisting with breast cancer [19, 20].

BPA, which is widely used to make epoxy resins and polycarbonate plastics, has weak estrogenic activity and belongs to the family of environmental endocrine disruptors (EEDs) [21, 22]. Animal research has shown that BPA can lead to precancerous lesions and carcinoma in the reproductive system and breasts [21, 23, 24]. The U.S. Environmental Protection Agency has defined a lowest observable adverse effect level (LOAEL) of 50 mg/kg/day for BPA in rat research [25]. But recent studies found that BPA can create a biological effect below LOAEL [26–28]. T. j. Murray et al [26] treated rats with BPA from the fetal period at gradient concentrations of 2.5, 25, 250, and 1000 μg/kg/day; the percentage of ductal hyperplasia in the mammary glands including carcinoma *in situ* (CIS) increased significantly, even at the lowest level of 2.5 μg/kg/day. BPA can bind to ERα. The ERα inhibitor ICI182 780 can block the proliferation effect of BPA [28]. Evidence of BPA contributing to breast and reproductive tumors is sufficient, but the effects of BPA on TC development and ERα expression are largely unknown. Several studies have found that excess iodine was regarded to have a potential carcinogenesis effect on the thyroid. This present study aimed to observe the effect of BPA on TC development, with or without basic KI treatment. In our research, we speculate that BPA could promote thyroid proliferation and increase the susceptibility to TC on the basis of iodine excess. ERα may be correlated with the biological effect of BPA on thyroid carcinogenesis.

## RESULTS

### Urinary iodine concentration of F344 rats

**Figure 1:**
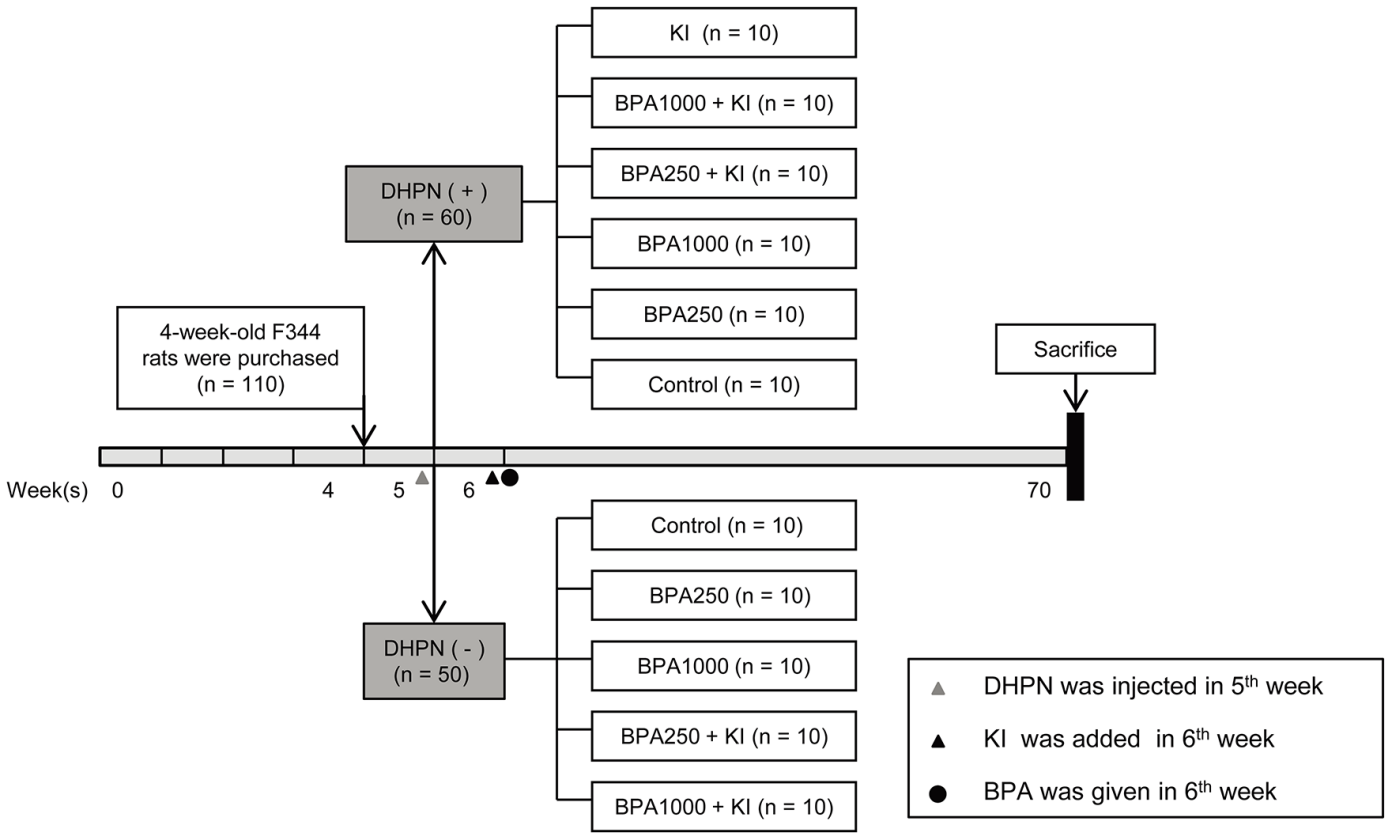
Schematic diagram of the experimental timeline 110 4-week-old female F344 rats were acclimated and divided into a DHPN (+) group (DA group, n = 60) and a DHPN (-) group (DN group, n = 50). One week later, KI 1000 μg/L was added to the drinking water. Then according to different dosages of BPA, the DN group was randomly divided into 5 subgroups: the control group, the BPA250 group, the BPA1000 group, the BPA250 + KI group, and the BPA1000 + KI group. The DA group was divided into 6 subgroups containing the aforementioned 5 subgroups and an extra KI group. The rats were sacrificed after 64 weeks of exposure.

As was shown in Figure 1, the rats were divided into several groups according to different treatments. To assess the iodine intake of rats, urinary iodine concentrations (UICs) in KI and the control of the DA groups were detected as 2 representative groups (the average daily water intake had no apparent difference among all the groups). The results showed the MUI in KI (320.36 μg/L) reached the standard of iodine excess recommended by WHO and was significantly higher than the control (86.09 μg/L) (*P* < 0.01). The average UIC in KI (311.84 ± 19.22 μg/L) was also significantly higher than in the control (90.50 ± 18.83 μg/L) (*P*< 0.001) (Table 1).

**Table 1:** Iodine intake and urine iodine concentration of rats

Group	n	Iodine intake (μg/d)	MUI (μg/L)	Average UIC (μg/L)
Control	5	5.20	86.09	90.50 ± 18.83
KI	5	20.50	320.36^**^	311.84 ± 19.22^***^

**P < 0.01, compared to the control group. ^***^P **<** 0.001, compared to the control group.

MUI: median of urinary iodine; UIC: urinary iodine concentration.

### General condition and bodyweights of F344 rats

To evaluate the effect of BPA on the rats’ growth process, their mental state, hair growth, and bodyweight were observed.

In the DN groups, the rats survived well; no apparent differences in living stage and hair growth were noticed among the BPA250, BPA1000, and controls, even on the basis of KI. As for bodyweight, BPA-exposed groups tended to decrease compared to the controls, but showed no significance. In the DA groups, negative mental states and thin hair were widely observed, but BPA did not worsen the general condition. Also, no obvious bodyweight changes occurred among all of the DA groups. Particularly, bodyweight in the DA control group (158.33 ± 17.84 g) was significantly lower than in the DN control group (198.80 ± 28.09 g) (*P* < 0.05) (Table 2).

**Table 2:** Data of bodyweight, serum FT_3_, FT_4_, and TSH concentration of rats

Group		N	Bodyweight (g)	**FT**_**3**_ **(pmol/L)**	FT_4_ (pmol/L)	TSH (μIU/mL)
DN						
	Control	10	198.80 ± 28.09^*^	3.49 ± 0.91	17.99 ± 2.70^*^	2.71 (1.78, 2.93)
	BPA250	7	156.33 ± 7.55	2.33 ± 0.52	20.18 ± 6.00	1.69 (1.40, 1.86)
	BPA1000	8	179.11 ± 16.70	3.48 ± 1.36	24.77 ± 10.12^*^	1.72 (1.56, 2.22)
	BPA250 + KI	9	160.27 ± 17.37	4.21 ± 0.67	20.42 ± 3.08	0.98 (0.61, 2.97)
	BPA1000 + KI	10	158.44 ± 34.54	2.99 ± 1.12	18.68 ± 6.09	1.57 (1.47, 1.67)
DA						
	Control	8	158.33 ± 17.84^*^	3.17 ± 0.38	18.37 ± 3.28	1.1 7(0.58, 4.16)*
	BPA250	9	158.00 ± 21.12	3.31 ± 0.74	21.26 ± 4.09	1.61 (1.53, 1.64)
	BPA1000	9	150.29 ± 10.01	2.85 ± 0.41	23.87 ± 3.94	2.13 (1.58, 2.68)
	BPA250 + KI	10	156.42 ± 17.43	3.37 ± 0.94	20.70 ± 2.18	1.51 (1.46, 1.59)
	BPA1000 + KI	10	162.50 ± 10.13	3.53 ± 0.68	18.44 ± 3.27	1.75 (1.61, 1.90)
	KI	9	157.22 ± 6.51	3.08 ± 1.06	19.63 ± 4.26	1.56 (1.48, 5.96)*

*Significant difference between the 2 groups at *P* < 0.05.

### Effects of BPA and KI on the absolute and relative thyroid weights of F344 rats

To assess whether BPA and KI could affect thyroid weights, the absolute and relative thyroid weights were recorded.

**Figure 2:**
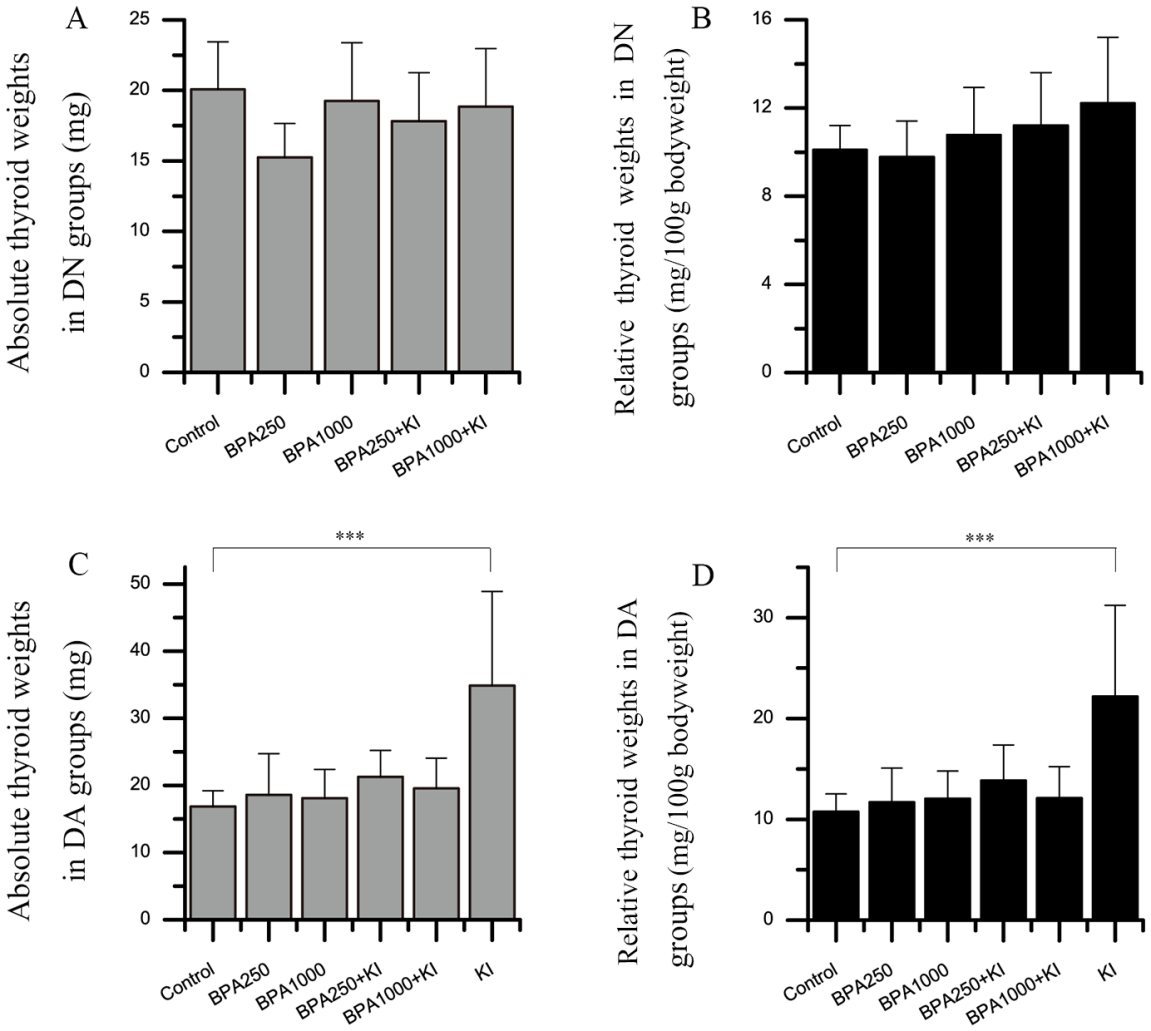
Effects of BPA and KI on the absolute and relative thyroid weights of rats Three samples were excluded due to their abnormally heavy weights (respectively, 235.0 mg in BPA250 + KI, 347.2 mg and 148.7 mg in BPA1000 + KI). **(A)** Absolute thyroid weights in the DN groups. **(B)** Relative thyroid weights in DN groups. The relative thyroid weights were in an increasing trend with the increased exposure dose of BPA and KI. **(C)** Absolute thyroid weights in the DA groups. **(D)** Relative thyroid weights in the DA groups. (^***^P < 0.001).

In the DN groups, the absolute and relative thyroid weights had no significant differences among groups (Figure 2A and 2B). With the increasing dosages of BPA and the administration of KI, relative thyroid weights showed a trend to increase, but had no statistical significance.

In the DA groups, both absolute and relative thyroid weights showed statistical significances among all groups (*P* < 0.01) (Figure 2C and 2D). BPA250 or BPA1000 alone did not apparently increase both absolute and relative thyroid weights. While the KI group had the heaviest thyroid weights and showed significant differences compared to the control group (34.84 ± 14.04 vs 16.87 ± 2.34 mg, *P* < 0.001; 22.17 ± 9.06 vs 10.75 ± 1.78 mg/100 g bodyweight, *P* < 0.001). As for the BPA250 + KI and BPA1000 + KI groups, 3 samples had abnormally heavy thyroid weights, respectively, 235.0 mg in the BPA250 + KI group, and 347.2 mg and 148.7 mg in the BPA1000 + KI group. After being excluded from the statistical analysis, the absolute and relative thyroid weights in the BPA250 + KI and BPA1000 + KI of the DA groups showed a weaker increase than the DA control group, but with no statistical significance.

### Effects of BPA and KI on incidence and pathological types of thyroid tumors

After a 64-week exposure time, a total of 11 thyroid tumors were found in all rats, 1 in the DN groups and 10 in the DA groups.

**Figure 3:**
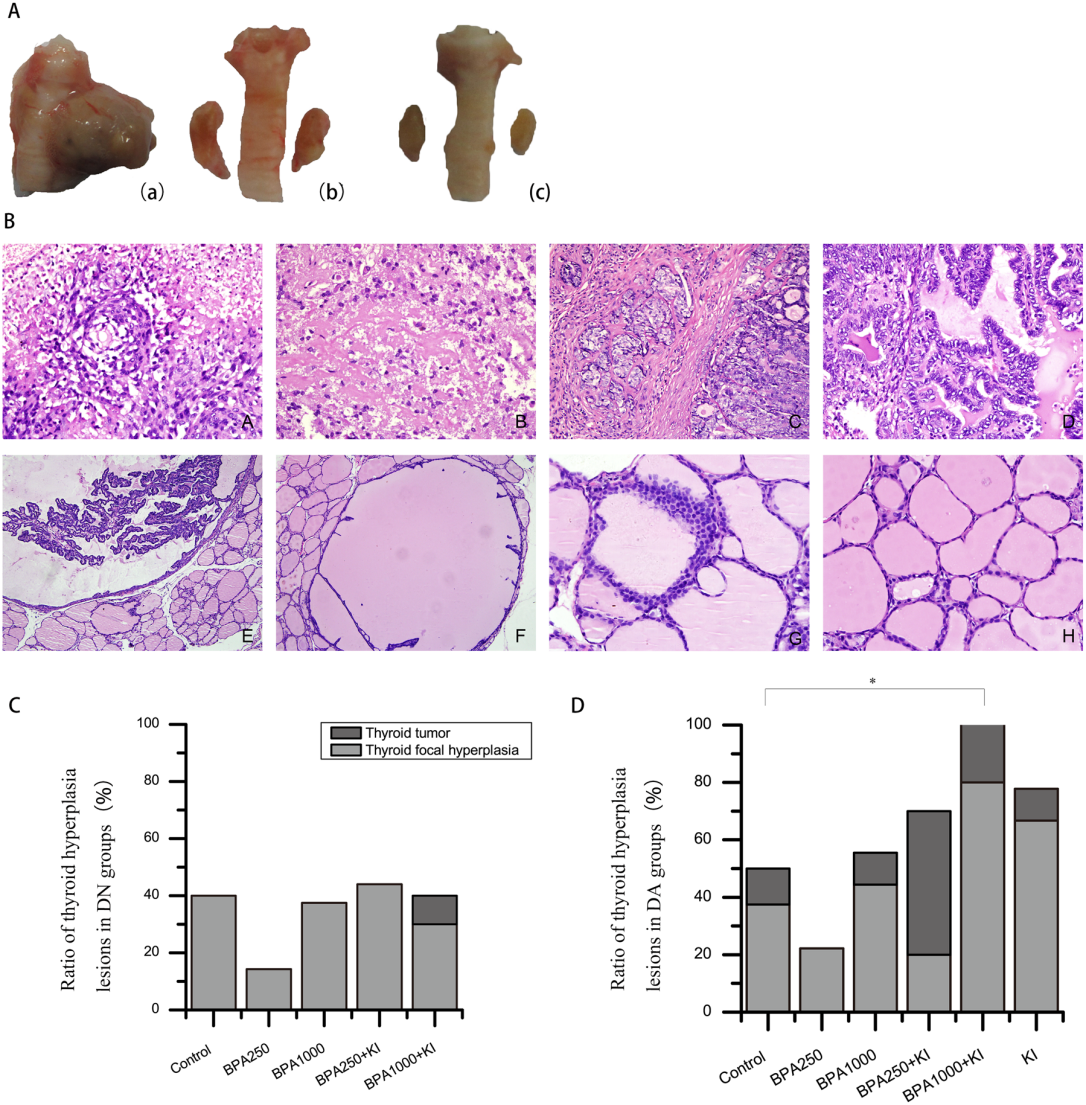
**(A)** Gross morphologies of the thyroid gland. (a): Typical PTC in BPA1000 + KI of the DA groups. (b): Thyroid hyperplasia in KI of the DA groups. (c): Normal thyroid in control of the DN groups. **(B)** Thyroid pathological structures under a microscope (H&E). (A): ATC (× 400) (B): MTC (× 400). (C): PTC with widely interstitial infiltration (× 200). (D): Classical PTC (× 400). (E): Atypical papillary hyperplasia (× 400). (F): Benign papillary hyperplasia (× 200). (G): C-cell proliferation (× 400). (H): Normal thyroid follicular follicles (× 400). **(C)** Ratio of thyroid hyperplasia lesions in the DN groups (including tumors and focal hyperplasia lesions). **(D)** Ratio of thyroid hyperplasia lesions in the DA groups. (^*^P < 0.05).

In the DN groups, 1 tumor occurred in BPA1000 + KI (1/10), which was a typical PTC. No tumors occurred in BPA250 or BPA1000. Tumor incidence had no significant differences among all groups (Figure 3C).

In the DA groups, BPA250 + KI had the highest tumor incidence of 50% (5/10); all were PTC, with interstitial infiltration in 1 tumor. Furthermore, 2 tumors occurred in the BPA1000 + KI group (2/10), an anaplastic thyroid carcinoma (ATC) and a medullary thyroid carcinoma (MTC), respectively. In addition, a PTC occurred in the control (1/8), a PTC accompanied by obvious interstitial infiltration occurred in the BPA1000 group (1/9), and a benign adenoma occurred in the KI group (1/9). No neoplasm occurred in the BPA250 group (0/9) (Figure 3D). Among all of the DA groups, tumor incidence showed no apparent differences. Typical TC pathological structures are shown in Figure 3B.

### Effects of BPA and KI on incidence and pathological types of thyroid hyperplasia lesions

In the present research, thyroid hyperplasia lesions were separated into 2 parts: thyroid tumors and focal hyperplasia lesions. Except for the thyroid tumors mentioned above, the numbers of thyroid focal hyperplasia lesions were also examined, including crowed cell arrangement, benign papillary hyperplasia, atypical papillary hyperplasia, and C-cell proliferation (Figure 3B). Particularly, atypical papillary hyperplasia and C-cell proliferation were respectively thought as precancerous lesions of PTC and MTC.

In the DN groups, the rate of thyroid focal papillary hyperplasia in BPA250 was a minimum of 14.29% (1/7) and in the BPA250 + KI group a maximum of 44.44% (4/9). No statistical significance was found among all DN groups (Figure 3C). In the DA groups, focal hyperplasia appeared most often in the BPA1000 + KI group, reaching 80% (8/10), followed by the KI group with a rate of 77.78% (7/9). BPA250 had the lowest rate at 22.22% (2/9). There also were no significant differences among all of the groups (Figure 3D). However, the total number of hyperplasia lesions (including both tumors and focal hyperplasia lesions) showed a significant difference among all of the DA groups (*P* < 0.05). Overall, 10/10 thyroids had a tumor or focal hyperplasia in BPA1000 + KI, which was significantly higher than in the control (4/8, *P* < 0.05), followed by KI (7/9) and BPA250 + KI (7/10) (Figure 3D). No statistical difference was found among all of the DN groups.

As for the pathological types, benign papillary hyperplasia and crowed cell arrangement happened in almost all of the groups with no clear regularity. In particular, 2 atypical papillary hyperplasia and 1 C-cell proliferation appeared in BPA1000 + KI of the DA groups, 1 atypical papillary hyperplasia happened in BPA250 + KI of the DA groups, and 1 atypical papillary hyperplasia and 1 C-cell proliferation occurred in KI of the DA groups.

### Effects of BPA and KI on the expression of PCNA

**Figure 4:**
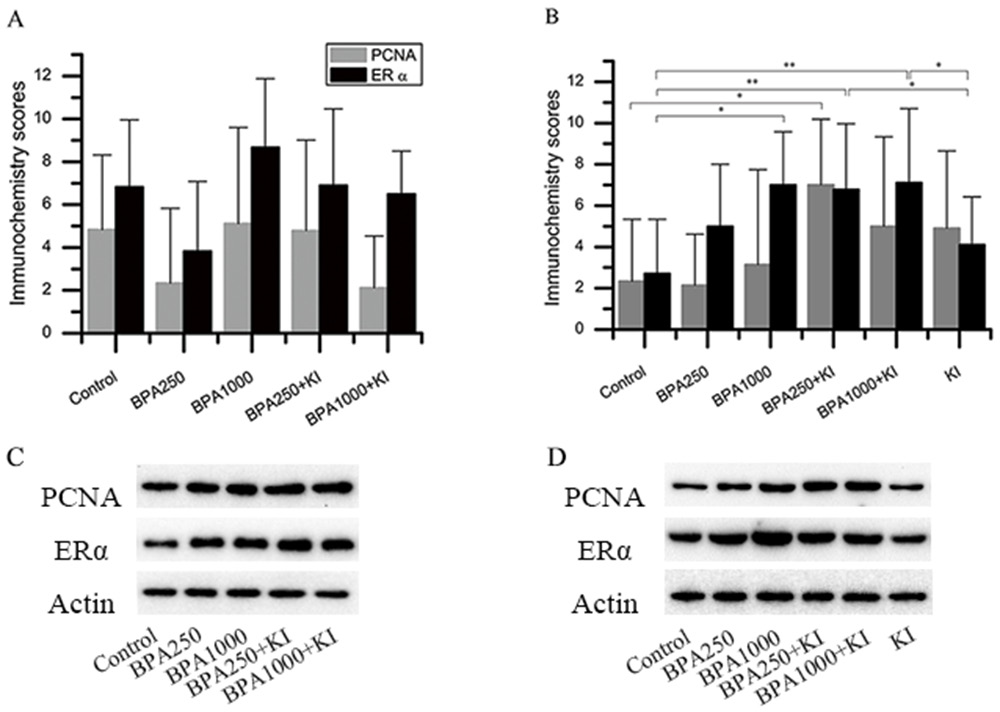
Immunochemistry and protein expression of ERα and PCNA in the rat thyroids **(A)** Immunochemistry scores of ERα and PCNA in the DN groups. **(B)** Immunochemistry scores of ERα and PCNA in the DA groups. **(C)** Protein expression of ERα and PCNA in the DN groups. **(D)** Protein expression of ERα and PCNA in the DA groups. (^*^P < 0.05; ^**^P < 0.01).

**Figure 5:**
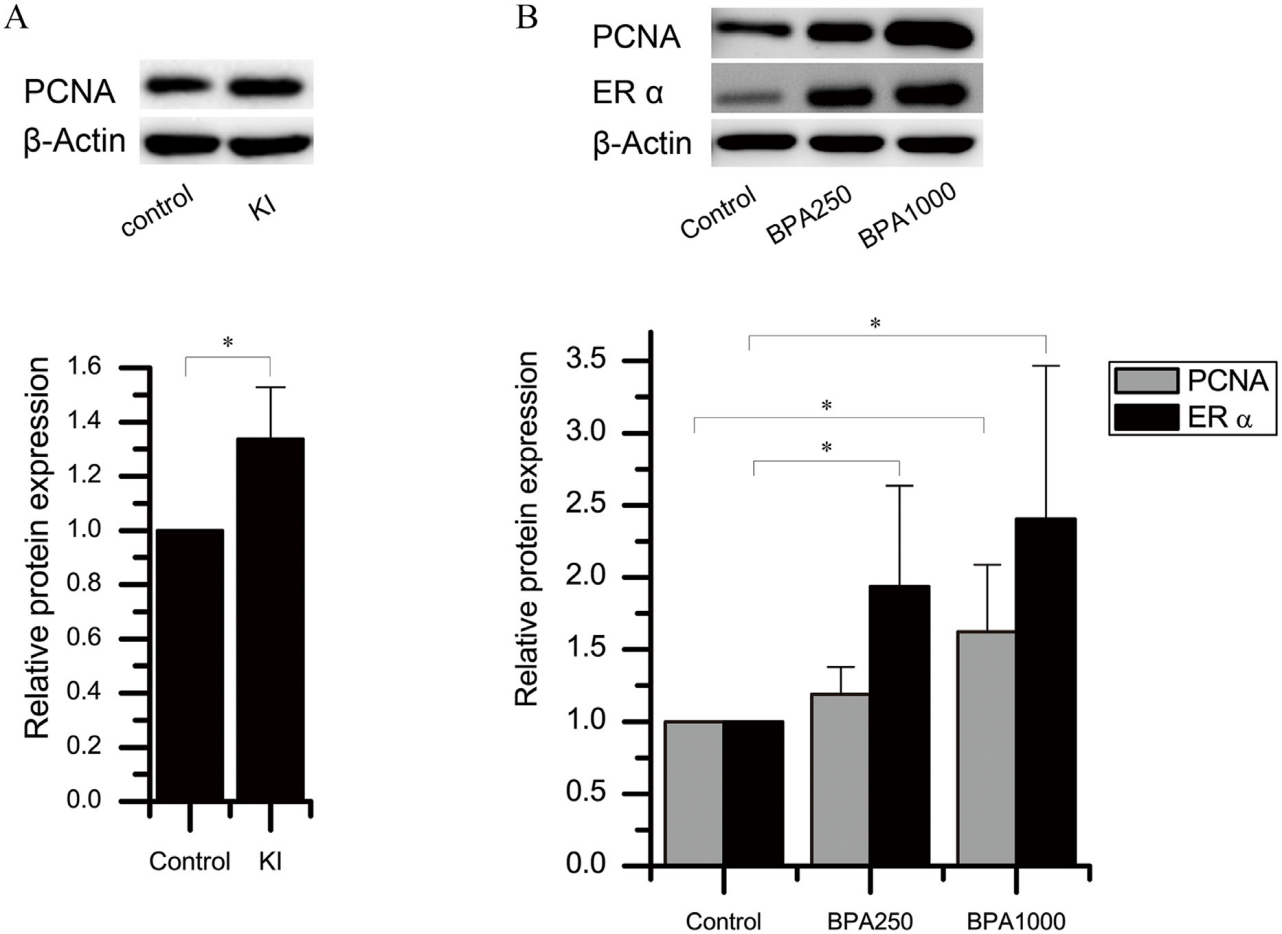
Protein expression of ERα and PCNA in the rat thyroids **(A)** PCNA protein levels in KI and control of the DA group. **(B)** ERα and PCNA protein levels of BPA 250, BPA1000, and control of the DN groups.*β*-actin is the loading reference. (^*^P < 0.05).

As for KI alone, the immunochemistry score of PCNA in KI of the DA groups had a tendency to increase, although there was no significance (Figure 4B). The Western blotting detection in the control and KI of the DA groups confirmed this tendency (*P* < 0.05) (Figure 5A).

Considering BPA alone, in the DN groups, the PCNA immunochemistry expression in BPA1000 was the highest among the control, BPA250, and BPA1000 groups, although no statistical significance was found among the 3 groups (Figure 4A). Consistently, in the DA groups, BPA1000 had the highest PCNA expression among the corresponding 3 groups (Figure 4B). The Western blotting result in the control, BPA250, and BPA1000 groups was consistent with the trend (Figures 4D and 5B).

When combining KI and BPA together, in the DN groups, PCNA immunochemistry expression in BPA250 + KI and BPA1000 + KI did not show an apparent increase than the other subgroups (Figure 4A). In the DA groups, PCNA expression in BPA250 + KI was the highest among all of the DA groups, which was significantly higher than control (*P* < 0.05), followed by BPA1000 + KI. No apparent difference was found between BPA250 + KI and BPA1000 + KI, as seen in Figure 4B. The Western blotting detection in each group showed similar tendencies (Figure 4C and 4D).

### Effects of BPA on the expression of ERα

In the DN groups, the immunochemistry score of ERα in BPA1000 tended to be the highest, followed by BPA250 + KI and BPA1000 + KI, but no significance was found among all groups (Figure 4A). In the DA groups, the immunochemistry scores of ERα showed significant differences among all groups (*P* < 0.05). ERα expressions in the BPA1000, BPA250 + KI, and BPA1000 + KI groups were significantly higher than in the control group (*P* < 0.05, *P* < 0.01, and *P* < 0.01, respectively). In addition, ERα expression in KI was significantly lower than BPA250 + KI and BPA1000 + KI (*P* < 0.05; *P* < 0.05) (Figure 4B).

Consistent with the increasing trend of ERα immunochemistry expression with the increasing dose of BPA, ERα protein expression also increased (Figure 4C and 4D). ERα protein expression in BPA250 and BPA1000 was apparently upregulated compared to the control group (*P* < 0.05; *P* < 0.05), while no significance existed between the 2 dosages (Figure 5B).

### The relationship between ERα and PCNA expression

#### Expression of ERα was correlated to PCNA

**Figure 6:**
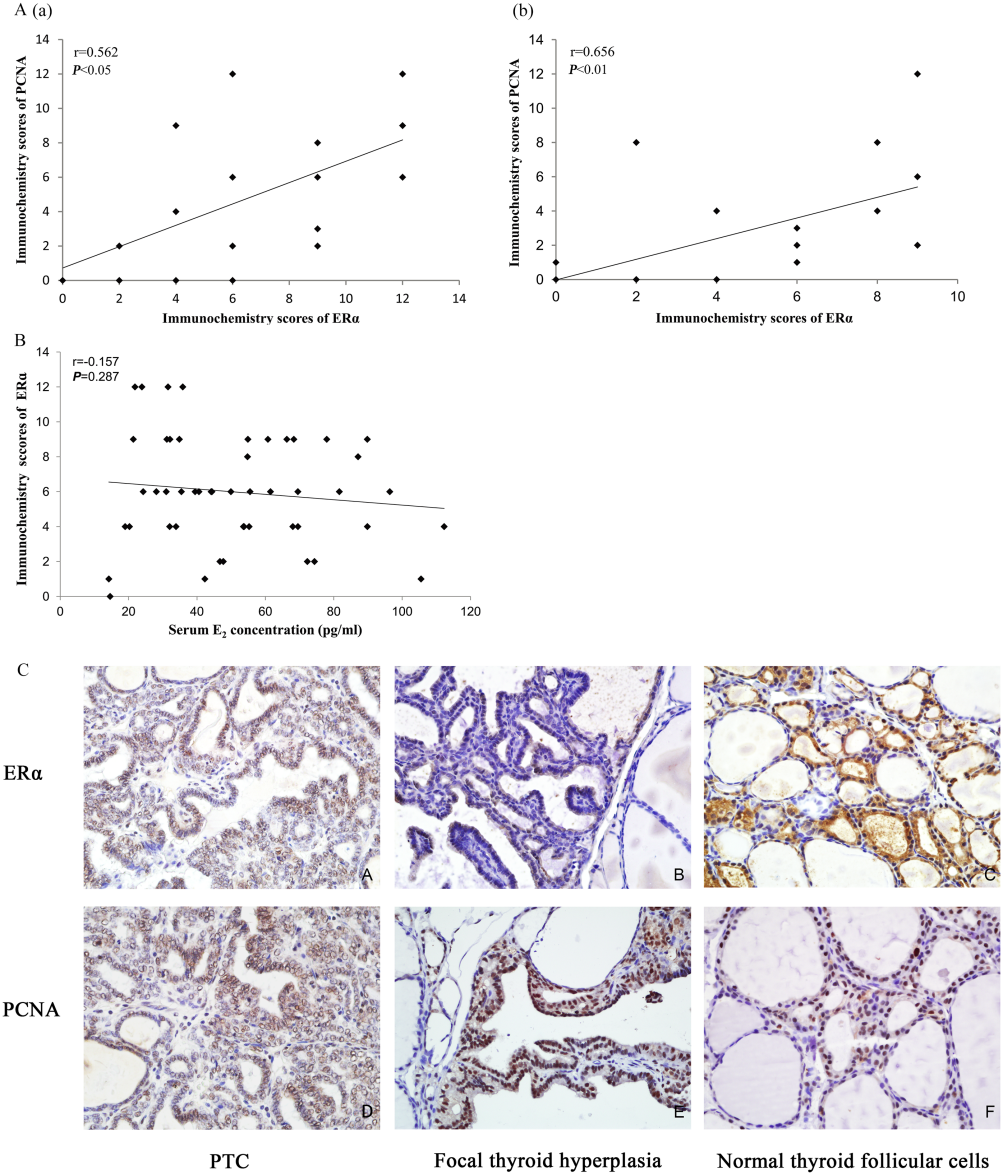
**(A)** Spearman’s correlation between the ERα and PCNA immunochemistry scores. (a): The correlation between ERα and PCNA in control, BPA250, and BPA1000 of the DN groups (r = 0.562; *P* < 0.05; n = 20). (b): The correlation between ERα and PCNA in control, BPA250, and BPA1000 of the DA groups (r = 0.656; *P* < 0.01; n = 18) (r = Spearman’s correlation coefficient). **(B)** Spearman’s correlation between the serum E_2_ concentration and the ERα immunochemistry scores (r = 0.157; *P* > 0.05; n = 48). **(C)** ERα and PCNA immunochemistry location in the rat thyroids. (IHC staining) (×400) (A): Positive expression of ERα on PTC cells. (B): Positive expression of ERα on a focal hyperplasia lesion, accompanied by negative expression in the surrounding normal cells. (C): Positive expression of ERα on cuboidal epithelial cells in normal thyroid tissue surrounded by flattened epithelial cells with negative ERα expression. (D): Positive expression of PCNA on PTC cells. (E): Positive expression of PCNA on a focal hyperplasia lesion with negative PCNA expression in the surrounding thyroid cells. (F): Positive expression of PCNA on cuboidal epithelial cells in normal thyroid tissue.

After excluding the influence of KI on PCNA expression, Spearman’s correlation analysis of immunochemistry scores of ERα and PCNA was conducted in the control, BPA250, and BPA1000 of both the DN and DA groups. In the DN groups, the ERα expression was correlated to PCNA (r = 0.562; *P* < 0.05; n = 20) (Figure 6A(a)). Consistently, in the DA groups, the correlation between the ERα and PCNA scores was significantly positive (r = 0.656; *P* < 0.01; n = 18) (Figure 6 A(b)).

#### Immunological location of ERα was consistent with PCNA

In the present thyroid samples, ERα and PCNA were expressed in both normal tissues and hyperplasia lesions. ERα was expressed mainly on the cell nucleus, with less on the cell membrane and cytoplasm. PCNA was located on the cell nucleus.

In normal thyroid tissues, ERα was distributed on small follicles composed of cuboidal or columnar epithelial cells with less colloid in the lumens, whose function was thought to be active rather than flattened epithelial cells with rich colloid in the lumens (Figure 6C(C)) (it developed in all animals regardless of the exposure elements). Similarly, PCNA had a higher expression on the cuboidal and columnar epithelial cells compared to the surrounding flattened epithelial cells (Figure 6C(F)).

ERα expression in the hyperplasia lesions was more higher than in the surrounding normal tissues, especially in the thyroid tumors (Figure 6C(A)) and complex papillary branches Figure 6C(B)) (regardless of the interventions). Consistently, the density and color depth of PCNA was more obvious in tumors (Figure 6C(D)) and focal hyperplasia lesions (Figure 6C(E)) than in the surrounding normal follicular cells, indicating an increased proliferative activity.

### 17-β estradiol (E_2_) had no influence on ERα immunochemistry scores

In order to judge and exclude the influence of endogenous E_2_ on ERα expression, serum E_2_ levels were detected. The data was presented as the X-axis of Spearman’s correlation analysis between endogenous E_2_ and corresponding ERα expression, as is shown in Figure 6B. There was no significant correlation between E_2_ concentrations and corresponding ERα immunochemistry scores (r = -0.157, *P* = 0.287, n = 48).

### Effects of BPA and KI on serum FT_3_, FT_4_, and TSH concentrations

Serum TSH, FT_3_, and FT_4_ concentrations were examined to observe the effect of BPA and KI on thyroid function (Table 2).

In the DN groups, TSH in the control was the highest, with no apparent fluctuation happening among the other groups. With the increase of BPA doses, FT_4_ concentration showed a tendency to increase. FT_4_ in BPA1000 (24.77 ± 10.12 pmol/L) was significantly higher than in the control (17.99 ± 2.70 pmol/L, *P* < 0.05). In BPA250 + KI and BPA1000 + KI of the DN groups, compared to the control, the FT_3_ and FT_4_ concentration had a tendency to increase with no statistical differences.

In the DA groups, TSH in KI was significantly higher than in the control (*P* < 0.05). The average FT_4_ concentration in BPA1000 was the highest (23.87 ± 3.94pmol/L). The FT_3_ and FT_4_ concentrations in BPA250 + KI and BPA1000 + KI were also higher than in the control, but no statistical differences were observed among the groups.

## DISCUSSION

In the present study, TC models were developed using female F344 rats exposed to BPA for 64 weeks with/without the basic treatment of KI and DHPN. BPA increased the susceptibility to TC on the basis of DHPN and KI. BPA or KI alone could not directly increase TC incidence. The increased expression of ERα probably participated in the proliferation effect of BPA.

The carcinogenesis effect of KI in human studies has always been controversial, but it has been reported to promote TC in animal research. In a 2-stage carcinoma study, KI at a concentration of 260 mg/L promoted the genesis of TC in DHPN-treated F344 rats after 26 weeks of exposure [2]. Boltze et al [29] fed rats iodine 10-fold higher than controls for 110 weeks, and the thyrocyte proliferation rate and thyroid adenomas increased. Inconsistently, our results showed that 64 weeks of exposure to 1000 μg/L KI failed to increase the obvious incidence of thyroid tumors. This may due to the shorter exposure time and smaller dosage compared to other studies. Although no obvious increase of TC incidence occurred, KI remarkably stimulated thyroid growth and proliferation. As shown, the thyroid weight in the KI group was the highest and the PCNA expression in the KI group increased more than in the control group. TSH is in particular an independent risk factor for thyroid tumors. The serum TSH level in the KI group in the present research was higher than in the control, which may directly contribute to thyroid proliferation [30].

As an endocrine disruptor, BPA could disturb biological functions and organ structures in extremely low concentrations. As was reported, 250 μg/kg BPA increased prevalence of intraductal hyperplasia in mammary of female rats at pup day 400 [31]. Except for the breast, BPA lower than 1000 μg/kg could induce benign and malignant hyperplasia lesions in the uterus and ovary of CD-1 mice [32]. In contrast to previous studies, our results showed that BPA 250 μg/kg or 1000 μg/kg alone did not apparently increase the incidence of thyroid tumors and focal hyperplasia lesions, although with the stimulation of DHPN. The thyroid weights also had no apparent changes. We reasoned that this inconsistency was probably due to different sensitivities of binding sites in different organs toward xenoestrogen. Besides, as the first to explore the influence of chronic exposure to low doses of BPA (lower than LOAEL) on thyroid proliferation, the present concentration might be not sufficient to induce pathological changes without other basic stimulus. Although there was no significant increase of tumors and focal hyperplasia lesions, the PCNA protein in the BPA-exposed groups had a tendency to increase, indicating a potential proliferation effect on thyroid cells.

It is believed that the combination exposure of environmental disruptors with mutagens induces TCs. For example, 4 Gy radiation apparently induced thyroid tumors in F344 rats with the existence of 10-fold iodine intake [29]. Combined exposure to excess soybean and iodine deficiency promoted thyroid tumorigenesis in DHPN-initiated F344 rats [33]. In the present study, when BPA and KI are taken together, the incidence of PTC in BPA250 + KI of the DA groups reached the highest at 50%, and the total incidence of thyroid hyperplasia lesions (including both tumors and focal hyperplasia lesions) in BPA1000 + KI of the DA groups reached 100%, including precancerous lesions such as atypical papillary hyperplasia and C-cell proliferation. The result first proved that BPA could apparently promote thyroid carcinogenesis in the basic treatment of KI and DHPN in F344 rats.

Although BPA1000 + KI did not promote PTC, the malignant degrees of MTC and ATC were much higher than PTC. The results demonstrated that, on the basis of KI and DHPN, a lower concentration of BPA could increase the incidence of PTC while a higher concentration of BPA could elevate the malignancy degree of tumors. In toxicology studies, nonlinear dose-response curves between the biological activity and the concentration have been commonly reported, such as an “S”-shape or an inverted “U”-shape curve [34, 35]. Therefore, we speculated that BPA250 μg/kg was more effective in promoting PTC than BPA1000 μg/kg. Besides, DNA microarray studies *in vitro* reported that increasing doses of xenoestrogen resulted in entirely different arrays of genes, so there should be not only a quantitative change in end points as the dose increases. Instead, entirely different types of effects could occur [36, 37]. But this needs further research *in vivo* with a wider BPA concentration range.

In the present study, contrary to the KI group, the FT_4_ level in BPA1000 of the DN groups tended to increase while TSH had a mild decrease. Zoeller et al [38] also found a significant increase in T_4_ levels in Sprague Dawley (SD) rats, which was in line with our results. In humans, a cross-sectional study with 3394 participants found that increased urinary BPA was related to elevated FT_3_ and decreased TSH concentrations [39]. Considering the decreased tendency of TSH and increased tendency of FT_4_ in BPA-treated groups compared to KI groups, we considered that BPA promotes thyroid hyperplasia lesions via other pathways rather than directly disturbing thyroid function.

ERs belong to the nuclear receptor family and mainly include 2 types: ERα and ERβ. ERα is thought to be associated with the promotion of thyroid proliferation while ERβ plays an opposite role [40]. Nuclear ERα can directly bind to genes and induce downstream transcription, while membrane ERα (mERα) can participate in the MAPK signaling pathway and the PI3K-AKT pathway so as to induce a proliferation effect [41, 42]. Case-control studies show that ERα expression in PTC tissues is significantly higher than in normal tissues. Thyroid tumors with positive ERα have a larger size and higher prevalence of local metastasis [19, 43]. ERβ downregulation was mainly associated with neck and lymph node metastasis and distant metastasis [44]. Furthermore, several studies found ERβ expression had no special significance in TC tissues. Therefore, we mainly focused on ERα to explore the proliferation effect induced by BPA.

ERα expression has always been tightly related to PCNA expression. Hao L et al [45] found PCNA and ERα mRNA expression level in pituitary of 12-week-old female F344 rats were both enhanced after BPA treatment. ERα inhibitor ICI182 780 decreased ERα and PCNA mRNA levels in uteri of ovariectomized SD rats [46]. ERα also regulates cell cycle by upregulating PCNA and Ki-67 and suppressing p53/p21 to promote MCF-7 cell proliferation [47]. Consistently, in the present research, ERα expression and the immunochemistry location were positively correlated with PCNA expression. Besides, E_2_ levels had no correlation with ERα expression in the present research, which excluded the influence of internal E_2_ concentration fluctuation on ERα. So we concluded that the upregulation of ERα in the thyroid probably participated in the proliferation process of BPA. Several studies also had similar findings. Xu et al [48] found that BPA could interfere with ERα signaling in the developing hippocampus in an ER-dependent manner. Neonatal BPA exposure disrupted meiosis progression during the first wave of spermatogenesis and increased the expression of ERα and PCNA in the developing testes [49]. However, few studies have explored the possible mechanisms of how BPA increases ERα expression. Some research has speculated that BPA increases ERα expression via the ERα-mediated signaling pathway, since ERα antagonist ICI 182,780 could prevent ERα upregulation *in vitro* experiments [46].

There are some limitations to this study. First, we did not perform extensive molecular research to explore the exact mechanism of how BPA acts on ERα *in vivo* and *in vitro*. As a matter of fact, the main finding of our present study was to demonstrate the phenomenon that BPA exerts the ability to enhance the susceptibility of TC under the condition of DHPN and excess iodine. The exact molecular mechanism of how BPA can increase ERα should be studied in future work. Second, we mainly performed a primary study to explore the potential role of ERα on the pathogenesis of BPA-induced TC. However, there are also some other mechanisms that might participate in the carcinogenesis of TC, such as autophagy, apoptosis, cell proliferation, among others. Therefore, our study would be improved by measuring the expression of markers such as LC3, Beclin, Bax, and Bcl-2, to name a few.

In conclusion, in our research, a low dose of BPA could increase the susceptibility of TC on the basis of excess iodine and DHPN in F344 rats. BPA or KI alone could not induce an obvious increase of TC incidence. ERα probably participates in the proliferation process.

## MATERIALS AND METHODS

### Animals and treatments

Specific-pathogen-free 4-week-old female Fischer 344 rats (F344 rats) obtained from Vital River China (Beijing, China) were housed 5 to a polypropylene cage with polypropylene plastic (PP) as bedding in an air-conditioned animal room (relative humidity 55 ± 5%, temperature 23 ± 2°C, and ventilation 18 times/hr, with a 12-h light/dark cycle) and given a basal diet and tap water ad libitum. In order to avoid extra intake of BPA, the rearing cages, water bottles, and rat bedding were all made of PP that did not contain BPA. All experiments were performed following the protocols of the Animal Care and Use Committee of Shandong University.

Potassium iodide (KI, 99% purity, Sigma-Aldrich, St. Louis, MO, USA) was dissolved in drinking water at a concentration of 1000 μg/L (the iodine concentration was calculated at 765 μg/L). The iodine content in the feedstuff was approximately 500 μg/kg and in the drinking water was 10 μg/L. The average food intake per rat was approximately 10 g/day and water consumption was 20 ml/day. The average iodine intake per animal was calculated at 20.5 μg/day in the KI groups and 5.2 μg/day in the control groups.

BPA (99% purity, Sigma-Aldrich, St. Louis, MO, USA) was dissolved in ethanol (< 0.01%) and administered by gavage at a daily dose of 250 μg/kg and 1000 μg/kg bodyweight, respectively, which was far lower than the LOAEL concentration of 50 mg/kg/day.

DHPN (Santai Labs, Inc., Jiangsu, China) was dissolved in saline as a vehicle and injected subcutaneously at a single dose of 2800 mg/kg.

### Experimental design

After 1 week of acclimatization, a total of 110 5-week-old rats were randomly divided into a DHPN-injected (DA) group (n = 60) and a non-injected (DN) group (n = 50). One week later, the DN group was randomly divided into 5 subgroups: a control group, a BPA 250 μg/kg (BPA250) group, a BPA1000 μg/kg (BPA1000) group, a BPA 250 μg/kg + KI 1000 μg/L (BPA250 + KI) group, and a BPA 1000 μg/kg + KI 1000 μg/L (BPA1000 + KI) group, with 10 rats in each group. The DA group was divided into 6 subgroups, containing the aforementioned 5 subgroups and an extra subgroup: KI 1000 μg/L (KI) group, with 10 rats in each group. The rats were necropsied after 64 weeks of exposure (Figure 1). Their thyroids were dissected, weighed, and preserved for further histological examination, immunohistochemical staining, and Western blotting analysis. Serums were collected for hormone detection. Urine was collected for 24 h for iodine concentration detection.

### Urinary iodine detection

Twenty-four h rat urine was collected into 5 ml glass tubes and stored at -20°C. The urinary iodine concentration method was based on the Sandell-Kolthoff reaction after ammonium persulfate digestion, which was recommended in 2006 by the Ministry of Health of the People’s Republic of China. The calibration curve ranged from 0 to 300 μg/L.

### Histopathological examination

The thyroids were fixed in 4% formaldehyde for 72 h, then embedded in paraffin and sectioned into paraffin slides. The slides were deparaffinized, rehydrated, and rinsed in PBS. Then the slides were stained with hematoxylin and eosin, dehydrated, cleared in xylene, and mounted with coverslips. The number and types of hyperplasia lesions including thyroid tumors and focal hyperplasia lesions were examined under a microscope.

### Immunohistochemistry staining

Immunohistochemical staining for ERα and PCNA was performed on all groups. Tissues sections were deparaffinized, rehydrated, and subjected to microwave antigen retrieval. Endogenous peroxidase was then blocked with 3% H_2_O_2_. Nonspecific antibody binding was blocked with 5% goat serum. Then the sections were incubated with primary antibody at 4°C overnight. Primary antibodies were respectively rabbit anti-PCNA (1:200) and rabbit anti-ERα (1:200) purchased from Abcam (Cambridge, MA, USA). The sections were incubated with the biotinylated secondary antibody for 1 h and washed in PBS. Color reactions were performed with DAB. Then the sections were counterstained with hematoxylin, dehydrated, cleared in xylene, and mounted with coverslips. Negative control sections were incubated with PBS. According to the intensity of the dye color and the number of positive cells, the scores of specimens were calculated.

### Western blotting detection

Western blotting analysis for ERα and PCNA was performed on all groups. The total protein was extracted, mixed with 5 × SDS-PAGE sample buffer at a 4:1 ratio, and denatured by heating the sample to 99°C for 10 min. After electrophoresis on SDS-PAGE, the proteins were separated. Then the target proteins were transferred to signed polyvinylidene difluoride (PVDF) membrane (Millipore, Bedford, MA, USA). After incubation with 5% skim milk, the membranes were incubated with primary antibody for 18 h at 4°C. The primary antibodies for target proteins were respectively rabbit anti-ERα (1:150) and anti-PCNA (1:200) obtained from Abcam. Then the membranes were incubated with horseradish peroxidase conjugated secondary antibody for 1 h at room temperature with agitation. Bands were detected using the FluorChem E system (Bio-Techne, San Jose, CA, USA). The data were expressed as the relative net intensity of the target protein/β-actin (ZSBIO, Beijing, China). At least 3 independent biological replicates were performed.

### Hormone detection

FT_3_ and FT_4_ were detected using an automated chemiluminescence immunoassay analyzer (ADVIA Centaur XP). The normal range of FT_3_ was 2.3-6.3 pmol/L and FT_4_ was 10.3-24.5 pmol/L. The serum TSH level was determined using a TSH ELISA kit (CUSABIO, Wuhan, China) based on the quantitative sandwich enzyme immunoassay technique, with a detection range of 0.6 μIU/ml-24 μIU/ml. The level of E_2_ was detected via an E_2_ ELISA kit (R&D Systems, Inc., Minneapolis, MN, USA), which is based on the competitive binding technique. The assay range was 12.30-3,000 pg/ml. Steps were performed according to the manufacturer’s instructions.

### Statistical analysis

All data and statistical significance were determined using SPSS statistic software (version 19.0, Chicago, IL, USA). Quantitative data were expressed as mean ± SD. The normality test and the homogeneity of variance test were applied. One-way ANOVA and the LSD test were used to determine differences among the groups. When the variance analysis conditions did not agree, the Kruskal-Wallis test was used. Qualitative data were analyzed using Fisher’s exact test. In addition, Student’s t-test was applied to determine urine concentration differences. Spearman’s correlation analysis was applied between ERα and PCNA immunochemistry scores, and between serum E_2_ concentration and ERα immunochemistry scores. Differences were considered statistically significant at *P* < 0.05.

## SUPPLEMENTARY MATERIALS FIGURES



## Abbreviations

BPA: Bisphenol A
DHPN: N-Bis (2-hydroxypropyl) nitrosamine
ER: estrogen receptor
KI: potassium iodine
MUI: median urinary iodine
PCNA: proliferating cell nuclear antigen
PTC: papillary thyroid carcinoma
TC: thyroid carcinoma
UIC: urinary iodine concentration
