# Supplementary material for: Low dose of Bisphenol A enhance the susceptibility of thyroid carcinoma stimulated by DHPN and iodine excess in F344 rats
Source: Oncotarget. 2017 Jul 22;8(41):69874–87. doi: 10.18632/oncotarget.19434 (PMC5642523; doi:10.18632/oncotarget.19434)
Supplement: Supplementary file 1 [file oncotarget-08-69874-s001.pdf]

Low dose of Bisphenol A enhance the susceptibility of thyroid carcinoma stimulated by DHPN and iodine excess in F344 rats

SUPPLEMENTARY MATERIALS

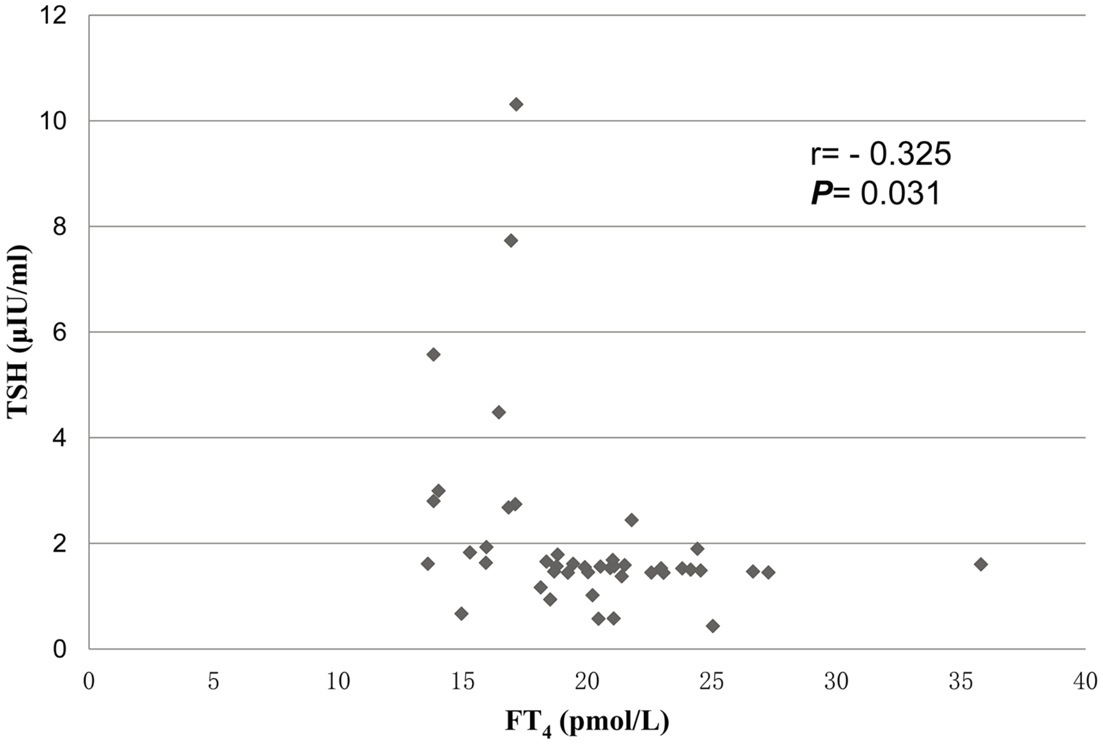

Supplementary Figure 1: The Pearson correlation between serum FT_4 and TSH concentration of F344 rats. (r = Pearson correlation coefficient).

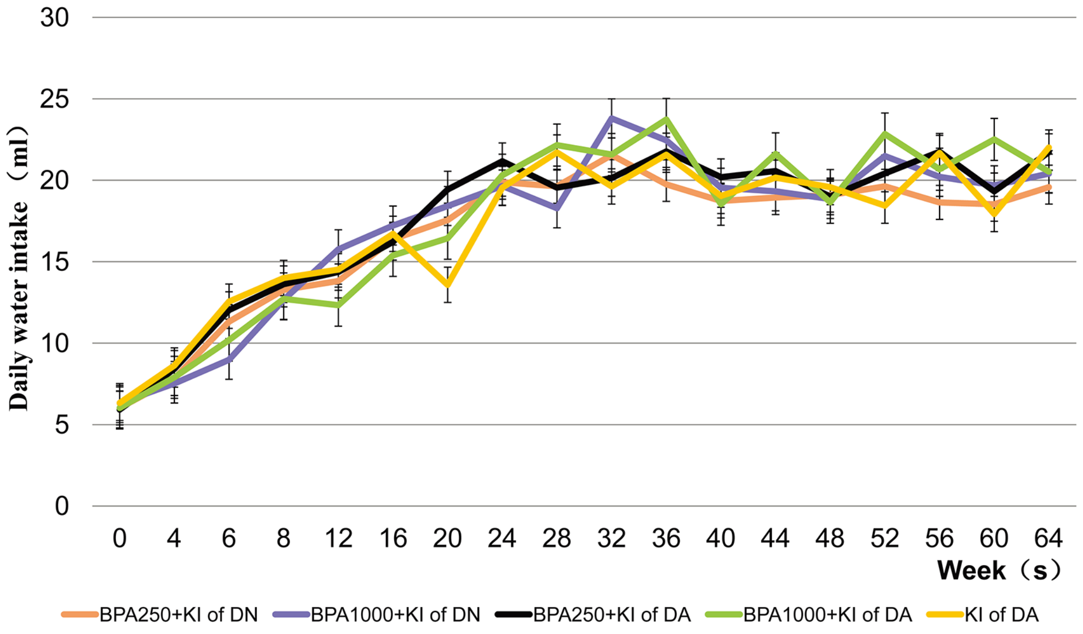

Supplementary Figure 2: The average daily water intake of F344 rats.
